# Molecular mechanisms in Alzheimer's disease and the impact of physical exercise with advancements in therapeutic approaches

**DOI:** 10.3934/Neuroscience.2021020

**Published:** 2021-03-19

**Authors:** Kiran Kumar Siddappaji, Shubha Gopal

**Affiliations:** Department of Studies in Microbiology, University of Mysore, Mysuru, 570006, Karnataka, India

**Keywords:** Alzheimer's disease, amyloid-beta plaques, dementia, neurofibrillary tangles, neuroinflammation, neurodegeneration, physical exercise

## Abstract

Alzheimer's disease (AD) is one of the most common, severe neurodegenerative brain disorder characterized by the accumulation of amyloid-beta plaques, neurofibrillary tangles in the brain causing neural disintegration, synaptic dysfunction, and neuronal death leading to dementia. Although many US-FDA-approved drugs like Donepezil, Rivastigmine, Galantamine are available in the market, their consumption reduces only the symptoms of the disease but fails in potency to cure the disease. This disease affects many individuals with aging. Combating the disease tends to be very expensive. This review focuses on biochemical mechanisms in the neuron both at normal and AD state with relevance to the tau hypothesis, amyloid hypothesis, the risk factors influencing dementia, oxidative stress, and neuroinflammation altogether integrated with neurodegeneration. A brief survey is carried out on available biomarkers in the diagnosis of the disease, drugs used for the treatment, and the challenges in approaching therapeutic targets in inhibiting the disease pathologies. This review conjointly assesses the demerits with the inefficiency of drugs to reach targets, their side effects, and toxicity. Optimistically, this review directs on the advantageous strategies in using nanotechnology-based drug delivery systems to cross the blood-brain barrier for improving the efficacy of drugs combined with a novel neuronal stem cell therapy approach. Determinately, this review aims at the natural, non-therapeutic healing impact of physical exercise on different model organisms and the effect of safe neuromodulation treatments using repetitive Transcranial Magnetic Stimulation (rTMS), transcranial Electrical Stimulation (tES) in humans to control the disease pathologies prominent in enhancing the synaptic function.

## 1. Introduction

More than a hundred billion long branching extensions of neurons form complex molecular connections between inter-neuron circuits through trillions of synapses in the adult human brain [1]. Electrochemical nerve impulses travel rapidly between neural circuits regulating sensations, language, memories, thinking, skills, feelings, emotions with control, and co-ordinated body movements [2]. Neurodegenerative brain disorders are characterized by damaged brain cells with loss of neuron connections, drastically affecting a person's ability to carry out daily activities, medically termed as, Dementia [3]. Globally, dementia is the fifth leading cause of death with a new case every three seconds. Currently, about 23 million in the Asia Pacific [4], 8.8 million in Europe [5], 5.8 million in the USA [6], 5.3 million in India [7], 50 million people worldwide are living with dementia and it would reach 152 million by 2050 [8]. According to the global survey from world Alzheimer's reports in 2020, the total estimated worldwide cost of dementia is one-trillion US $ and would reach two-trillion US $ by 2030 [9].

AD is a type of progressive neurodegenerative disorder and the most common cause of dementia in aging groups [10]. The first case was reported in 1906 by Dr. Alois Alzheimer, a German physician who identified the typical disease of the brain in a 51 years old woman patient; Auguste Deter with series of devastating symptoms like trouble in understanding, planning, execution of the task, changes in mood, psychological worsening, and profound memory loss [11]. Autopsy of the brain revealed pervasive atrophy of the cerebral cortex, brain tissues stained with silver salts showed distinct dense deposits of neuritic plaques [12]. Accumulation of abnormal protein aggregates or amyloid-beta (Aβ) plaques in the synapse region between communicating neurons interferes with nerve impulse transmission and leads to neuron death [13]. The internal supporting structure of a neuron is made up of integrated tubular microtubules, bridged by tau-proteins that stabilize the neuron [14]. Tau is an important cytoskeletal protein that maintains the structural stability of neurons associated with microtubule assembly, helps in nutrient transport [15].

Clinical aspects of Dementia are categorized into three stages, the early or mild stage with deterioration in learning and memory, language difficulties, declined vocabulary fluency, execution of functions, changes in personality and behavior, problems in olfactory and other sensory perception, loss in episodic, semantic, and implicit memories, depression with Mild Cognitive Impairment (MCI). Moderate or middle stage with progressive neuropsychiatric loss of reading, writing skills, difficulty in speech with incorrect word substitution, wandering, anxiety, irritability, aggression, agitation, delusion, sundowning, and loss of long-term memory. Severe or late-stage characterized by loss of speech with language reduced to phrases or only words, lack of emotion, fatigue, and decline in muscle mass with complete dependency on caregivers [16],[17]. Dementia is caused by multiple etiological conditions like morphological modification in the brain with narrowed small blood vessels and declined vascular density followed by endothelial dysfunction which leads to vascular dementia. Small blood vessel narrowing in the brain is termed arteriosclerotic dementia. Decreased cerebral blood flow in the brain leads to the oxygen-depleted hypoxic condition. Inflammation of vessels in the brain causes vasculitis with ischemic hemorrhage and stroke. The molecular events of vascular dementia are closely associated with dementia caused by AD pathologies—Aβ plaques and neurofibrillary tangles. In turn, the accumulation of abnormal α-synuclein protein aggregates to form Lewy bodies in the neurons with brain atrophy causes dementia in Parkinson's Disease (PD). The atrophy of the frontal and temporal lobe in the brain causes frontotemporal dementia [18]. Neural loss, gliosis, spongiosis, neuroinflammation, impairment in language, physical inactivity, cognitive impairment, stress, hypertension, confusion, irritation, depression, and severe memory loss are the overall complex characteristics of dementia [19].

This review focus on the different molecular hypothesis of tau forming neurofibrillary tangles (NFTs), amyloidogenic processing of amyloid precursor protein in forming amyloid plaques, interconnected molecular events involved in neuroinflammation and neurodegeneration. This review sheds light on the factors influencing dementia, types of biomarkers available for the diagnosis of AD, activity, and toxicity of various drugs. As a measure of habitual curative, the vital effects of physical therapies and the outbreaks of research in divergent model organisms successful in decreasing the Aβ plaques, tau phosphorylation, NFTs, neuroinflammation in the brain put forward ideas with multiple strategies on controlling disease pathologies and future therapeutic perspectives in dealing with AD [20].

## 2. Molecular mechanisms in AD

### 2.1. Tau hypothesis

**Figure 1.**
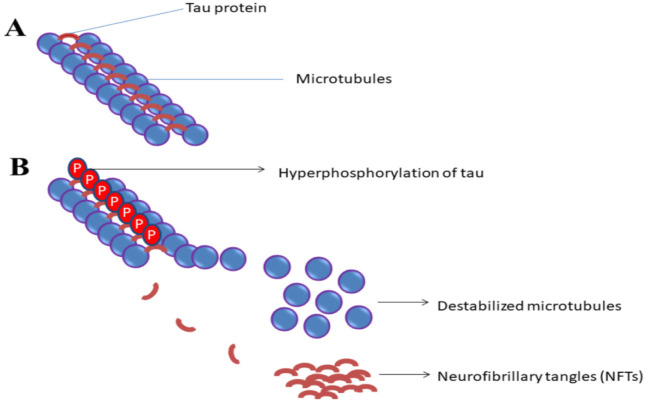
Role of Microtubules, tau proteins in neuron stability, and hyperphosphorylation in neural disintegration. (A) Microtubules and tau protein together stabilize structural integrity in a healthy neuron. (B) Hyperphosphorylation of tau leads to dissociation of tau proteins and destabilization of microtubules from the neuron. The dissociated tau filaments pairs to form NFTs, structural integrity of the neuron are lost in the disease state.

Disaggregation of tau proteins forms NFTs inside the neurons and blocks the transport of nutrients lead to neurodegeneration, termed tauopathy [21],[22]. In the AD state, microtubules dissociate from tau proteins by chemical changes due to hyperphosphorylation and pairs with another tau protein threads forming NFTs involving the mechanism of the tau hypothesis (Figure 1) [23].

Aβ neuronal plaques and NFTs are the gold standard hallmarks of AD that develop gradually and spreads in the hippocampus and cerebral cortex regions of the brain [24]. Pathophysiology of the AD brain characterizes degeneration of neurons in the frontal, temporal, parietal lobes of the cerebral cortex, with cingulate gyrus and certain subcortical regions influencing synaptic dysfunction [25]. Due to cortical atrophies in AD, the gyri-folds of the brain diminish and the sulci-spaces in the folds of the brain are grossly enlarged. In AD state, the area of the brain shrinks in invariably in size different from that of a normal brain [26].

### 2.2. Molecular mechanism in the brain at normal state

**Figure 2.**
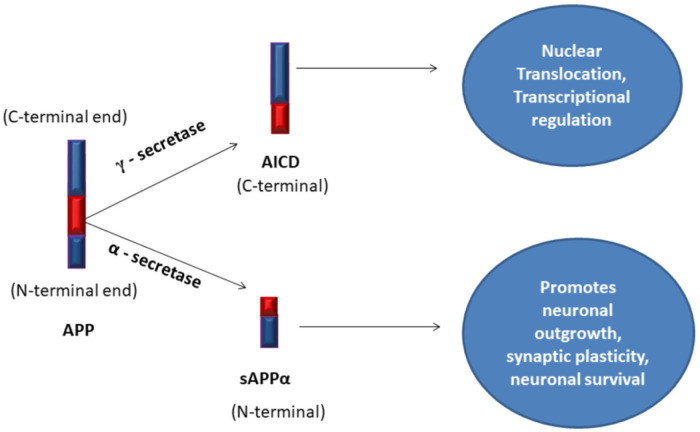
Non-amyloidogenic-Amyloid Precursor Protein (APP) processing by α-secretase and γ-secretase in a healthy neuron.

A larger transmembrane protein Amyloid Precursor Protein (APP) present on the neuronal membrane is involved in the growth, development, survival, and repair of neurons [27]. APP has two terminal ends spanning the neuronal membrane; N-terminal and C-terminal. An enzyme, α-secretase recognizes and cleaves the C-terminal end of APP near the cell surface to generate soluble Amyloid Precursor Protein alpha (sAPPα), which helps for neuronal plasticity and outgrowth of neurons [28]. sAPPα is further cleaved by an enzyme, γ-secretase to generate a secreted fragment called AICD (Amyloid Precursor Protein Intra Cellular Domain), which helps for neuronal transcription regulation and translocation [29]. Non-amyloidogenic APP processing (Figure 2) by α-secretase and γ-secretase sequentially is the scenario of events in the brain at normal conditions [30].

α-secretase is the first enzyme that initiates the pathway of events, called the α-secretase pathway. In the normal state, the receptors on neurons are free to accept ligands for normal nerve impulse transmission. The glucose transporters on the neuron transport glucose, N-Methyl-D-Aspartate Receptor (NMDAR), α-Amino-3-hydroxy-5-Methyl-4-isoxazole Propionic acid Receptor (AMPAR) transport Na^+^, Ca^2+^ for maintaining homeostasis of synaptic transmission [31]. Nicotinic Acetylcholine Receptors (nAChRs) and muscarinic Acetylcholine Receptors (mAChRs) binds to acetylcholine neurotransmitters helping neurotransmission between communicating neurons [32],[33].

### 2.3. Molecular mechanism in the brain at AD state

**Figure 3.**
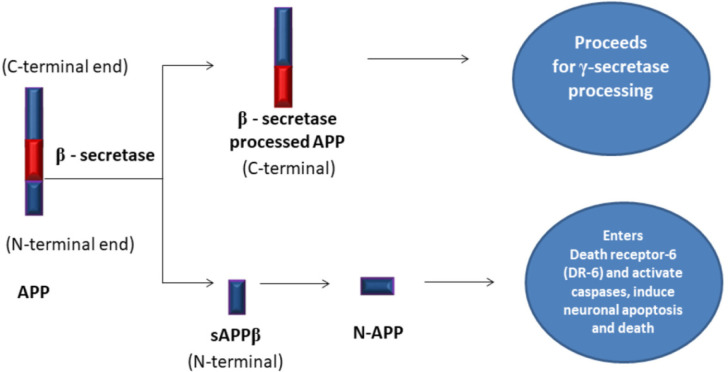
Amyloidogenic-Amyloid Precursor Protein (APP) processing by β-secretase in AD state.

In the AD state, instead of α-secretase, an enzyme β-secretase or β-Amyloid Cleaving Enzyme-1 (BACE-1) performs amyloidogenic-APP processing from the C-terminal end to form soluble Amyloid Precursor Protein beta (sAPP β). Eventually, sAPPβ enters death receptor-6 on the neural membrane and activates caspases (caspase-6). The activated caspases initiate apoptotic pathways and induce neural death (Figure 3) [34].

The remaining membrane-bound APP is further recognized by an enzyme, γ-secretase generates Aβ monomer fragments of (Aβ40/Aβ42) 40–42 amino acids length [35]. Out of which, Aβ40 is majorly formed, leaving only around 10% Aβ42. Many monomers of Aβ peptide aggregates form dense, insoluble oligomers or senile plaques [36]. Amyloid hypothesis comprises the cleavage of APP from BACE-1 to form Aβ-peptides. The misfolded peptides formed are different in conformation and are released from donor neurons to the extracellular space either as naked protein or vesicles as exosomes taken up by recipient neurons through receptor-mediated endocytosis [37]. The formed Aβ40/Aβ42 accumulates on glucose transporter receptor [38], NMDAR [39], AMPAR [40], nAChR [41], mAChRs [42] causing impairment in synaptic transmission by blocking ion channels and neurotransmitters through calcium dysregulation [43]. The formation of Aβ plaques could be linked to preceding cortical tau pathology, but Aβ independent regulators like Apolipoprotein-E (ApoE), cholesterol metabolism, receptor-mediated endocytosis, and microglial activation may induce tau pathology [44].

**Figure 4.**
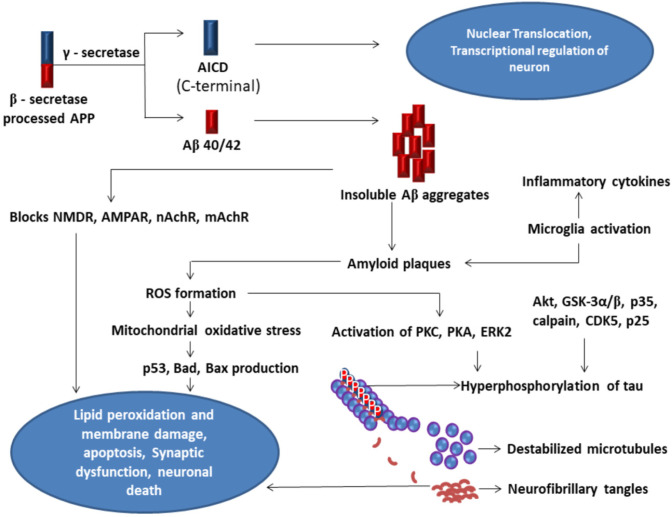
Multiple molecular factors involved in neural death—Post to β-secretase processing the remained C-terminal APP-transmembrane protein is recognized and cleaved by γ-secretase to form AICD which helps neuronal regulation by transcription and translocation. Cleavage by γ-secretase also generates Aβ short peptide of 40–42 amino acids length (Aβ40/Aβ42). Monomers of Aβ peptide form Aβ clumps of amyloid plaques. The formed amyloid plaques block NMDAR, AMPAR, nAChR, mAChRs on the neuronal membrane and impair synaptic transmission. Aβ plaques induce mitochondrial oxidative stress by the generation of ROS and activate series of caspases with the production of p53, Bad, Bax inducing apoptosis with neuron death. Amyloid plaques also activate inflammatory cells like astrocytes and microglia to produce chemokines and cytokines to induce inflammatory reactions. ROS formed by Aβ activatesPKC, PKA, ERK2, and hyperphosphorylates tau to disintegrate leads to disassociation of microtubules, forms clumps of NFTs. Activation of PKB or Akt to activate GSK3α/β, P35-Calpain mediated activation of CDK5 and P25 also induces hyperphosphorylation of tau to form NFTs, all together ultimately leads to neural death.

Aβ plaques formed in the neurons activate microglia and astrocytes that produce chemokines and cytokines involved in the formation of Reactive Oxygen Species (ROS) [45]. This creates mitochondrial oxidative stress and activates series of apoptotic caspases by the production of p53, Bad, and Bax production leading to lipid peroxidation, membrane damage, and neuronal death [46],[47]. ROS formed by Aβ activates—protein kinase C (PKC), protein kinase A (PKA), and Extracellular Signal-Regulated Kinases2 (ERK2), induces hyperphosphorylation of tau and destabilizing microtubules forming NFTs [48]. Hyperphosphorylation of tau is also mediated by activation of protein kinase B (PKB) or Akt to activate Glycogen Synthase Kinase 3α/β or GSK3α/β [49]. P35-Calpain mediated activation of Cyclin-Dependent Kinases 5 (CDK5) and P25 also induces hyperphosphorylation of tau to form NFTs and neuronal death (Figure 4) [50].

Regulation of memory and learning in the brain is controlled by the cholinergic neurotransmitter system with AcetylcholinEsterase (AchE) enzyme found in neuromuscular junction acts through hydrolytic activity [51]. Acetylcholine is an important neurotransmitter required for regular synaptic transmission [52]. AchE degrades acetylcholine into choline and acetate terminating a synaptic transmission. Degeneration of the cholinergic system is an observed mechanism in AD, where the levels of acetylcholine are drastically reduced by the activity of AchE [53].

### 2.4. Integrated connection of mitochondrial oxidative stress and inflammation with Aβ

Aging cell degenerates by free radicals generated through oxidative imbalance forming ROS [54]. Enzymes like catalase, superoxide dismutase, glutathione peroxidase combat ROS by antioxidant defense mechanism [55]. If anti-oxidant enzyme levels decrease or levels of ROS exceed the toxic threshold due to overproduction, it induces oxidative stress in a cell [56] leading to mitochondrial dysfunction which is a common phenomenon in aging diseases like AD, PD, and other neurodegenerative disorders [57]. Mitochondria, being the powerhouse of the cell produce a majority of ATPs by coupling electron transfer to the pumping of protons across the inner mitochondrial membrane [58]. In certain cases, electrons escape the Electron Transport Chain (ETC) and cut oxygen to ROS like ·O_2_, ·OH, and H_2_O_2_ causing oxidative damage [59]. Brain cells use more than 20% of the oxygen consumed by the body and form a high rate of ROS during oxidative phosphorylation [60].

Aging-associated AD causes early cellular changes in the mitochondria causing dysfunction. Aβ enters mitochondria, induces oxidative stress with free radicals, damages mitochondrial membrane, decreases cytochrome oxidase activity, blocks mitochondrial protein transport, alters ETC, and inhibits ATP production. Aβ aggregates in the synaptic terminals degenerate neurons, blocks neural circuits, and lead to cognitive dysfunction [61]. Employing mitochondrial therapeutic approaches on using natural and targeted anti-oxidants for scavenging free radicals maintains mitochondrial functioning with enhanced ATP production, decreases lipid peroxidation, and protects neurons from oxidative stress [62]. Studies using cDNA microarray in APP-Tg2576 transgenic mice model for gene expression related to mitochondrial energy metabolism and apoptosis showed up-regulation in 2-months aged-Tg2576 mice, 5 & 18-months aged-Tg2576 mice. *In situ* hybridization confirmed the mitochondrial genes-ATPase-6, heat shock protein-86 (HSP-86), programmed cell death gene-8 (PCD-8) are up-regulated in Tg2576 mice compared to wild type mice, which correlates with mutated APP/Aβ-induced mitochondrial energy metabolism impairment, oxidative damage in the neurons of hippocampal regions, and cerebral cortex [63]. Removal of damaged mitochondria from the cell is a normal process called Mitophagy. Excessive Aβ, p-tau and ROS production induces impaired autophagy and mitophagy in aging and AD [64]. Dynamin-related protein1 (Drp1) are natural enhancers that regulate mitochondrial fission and induce mitophagy. Drp1 is synergistically associated with Aβ and p-tau inducing abnormal mitophagy in AD, inhibited Drp1 levels up-regulate normal mitophagy in AD [65]. Formation of NFTs due to hyperphosphorylation and destabilization of tau from microtubule assembly is associated with oxidative stress, lipid peroxidation, decreased Insulin-like Growth Factor-1 (IGF-1), Aβ oligomers mediated by astrocytes, intraneural Aβ accumulation, impaired axonal transport, caspases activated mitochondrial apoptosis, reduced ATP synthesis and synaptic dysfunction [66].

The brain is rich in lipids with unsaturated fatty acids, being easy targets for lipid peroxidation. It has a weak antioxidant defense mechanism compared to other organs with fewer ROS detoxifying enzymes compared to the liver and kidney [67]. A higher level of iron catalyzes the formation of ROS in certain areas of the brain [68]. Glutathione, tri-peptide functions in defense against reactive oxygen species by detoxifying ROS and reduction of peroxides [69]. Oxidative damage caused by Aβ induces toxicity in the cerebral region of the brain by inhibition of mitochondrial enzymes like α-ketoglutarate dehydrogenase, cytochrome c oxidase, and pyruvate dehydrogenase leading to reduced levels of mitochondria [70]. The formation of Aβ plaques and NFTs induces activation of inflammatory cells such as astrocytes & microglia to secrete pro-inflammatory cytokines like Tumour Necrosis Factor-α, Interleukin-6 (IL-6), and anti-inflammatory intermediates [71]. Astrocytes activated by Aβ also release chemokines and cytokines including IL-β, Nitrous Oxide Synthase-2 (NOS-2) [72]. Between neurons and glial cells, chemokines act as messengers and attract microglia to further secrete pro-inflammatory mediators which collectively induce neuronal damage [73]. A nuclear transcription factor called Nuclear Factor Kappa B (NF-κB) regulates many mechanisms including cell growth and development, cell survival, inflammatory responses, and apoptosis [74]. Aβ has an impact to increase NF-κB activity in brain cells with degenerating neurons [75]. Death of neurons may take place either by inflammation of neurons called necrosis or by hyperactivation of apoptosis induced by death caspases [76]. Unfortunately, the loss of neurons in the adult brain cannot be compensated by the generation of new neurons [77].

## 3. Risk factors influencing dementia in AD

### 3.1. Impact of obesity, hyperinsulininsm, diabetes & cardiovascular diseases in dementia associated with AD

Obesity and dementia are closely associated, the fat cells in the brain release adipose secreted proteins with inflammatory cytokines induces increased blood supply, damages white matter in the brain, and causes vascular dementia with cognitive impairment, increases the risk of AD [78]. Diet rich in carbohydrates and saturated lipids is one of the major causes of obesity which affects insulin secretion and glucose metabolism in the cerebral region of the brain. Oxidative stress, impaired insulin secretion, activation of inflammatory cytokines is the common intracellular mechanisms in Type-2 Diabetes and AD [79]. Insulin is a hormonal protein regulating blood sugar level which is also transported from the peripheral nervous system to the brain, especially essential for cognitive development of the brain. In insulin-deficient conditions, the risk for obesity increases, and insulin uptake by the brain declines which leads to dementia [80]. The expression of the level of insulin and its receptors decline by normal aging and still diminish in AD [81].

Combined factors with age and obesity, elevated insulin levels are associated with activation of neuro-inflammatory signaling pathways and co-laterally increase amyloid-beta deposits in the brain causing AD [82]. As a common strategy, routine physical activity and a disciplined dietary lifestyle with control in intake of fat and carbohydrates may be the key to reduce the risk of complications associated with insulin resistance, diabetes, and AD. Obesity and stress with high blood pressure induced hypertension are interconnected in causing endothelial dysfunction, increasing cardiovascular morbidity with atherosclerosis and ischemic white and grey matter atrophy with inadequate blood flow to the brain, accumulation of amyloid plaques leading to cognitive dysfunction with encephalopathy and dementia [83],[84]. The dietary lifestyle and smoking habits impact metabolic function and increase the risk of association between diabetes and dementia [85].

Brain MRI studies in patients with diabetes are two-fold more prone to develop brain atrophy with a high risk of developing AD as compared to normal without diabetes [86],[87]. Studies on rat model (BBZDR/Wor) with diabetes revealed loss of neurons, neural dystrophy, elevated amyloid levels, and tau hyperphosphorylation with decreased expression of insulin and IGF-1 receptors [88]. Zinc-binding metalloprotease Insulin Degrading Enzyme (IDE) binds to substrates insulin and amyloid-β with more affinity for insulin, predictable to be associated with the interconnection between diabetes and AD. Studies in the transgenic AD mice models fed with a high-fat diet reported impaired insulin signaling with diminished IDE levels and increased amyloid-β levels [89]. The loss of IDE function may induce hyperinsulinemia correlated with insulin resistance and altered glucose tolerance, but the exact underlying mechanisms remain unclear. IDE inhibition by drugs showed a reduction in the degradation of insulin and amyloid-beta [90]. Hyperinsulinemia in diabetes resulted in suppressed degradation of amyloid-beta by IDE which further induces Aβ deposition in the brain [91]. Chronic hyperinsulinemia alters the insulin signaling pathway and increases insulin resistance in the brain with reduced uptake of insulin transport across the blood-brain barrier (BBB) induces cognitive dysfunction with dementia [92]. Diabetes and AD are closely related to many characteristic risk factors which include vascular parameters like stress, hypertension, anxiety, high blood pressure, cerebrovascular diseases, endothelial function, hypercholesterolemia, lipoprotein receptors, and oxidative stress. Lipoprotein receptor-related protein-1 (LRP-1) is a key signaling protein involved in metabolic defects and neurodegenerative diseases including AD. Studies in rats have shown improvement in Aβ-induced learning and memory impairments with functional LRP-1 regulating signaling pathways for Aβ clearance from the brain [93],[94]. The study reported an inverse in dementia by regulation of amyloid beta-processing and synaptic plasticity in the hippocampus of the brain monitored by a unique Leptin hormone secreted by adipose cells and enterocytes in the small intestine. Leptin acts as a neuroprotective hormone, inhibits the activity of the BACE enzyme, activates Aβ degradation, inhibits GSK3β, inhibits oxidative stress, and inhibits long-term potentiation and depression. Obesity in middle age groups with Leptin resistance increases the risk for developing AD [95].

### 3.2. Genetic aspects of dementia in AD

Mutations in multiple genes cause autosomal early-onset and late-onset familial AD in more than 1% of cases of AD. APP-coding gene located on chromosome 21q21 exhibit majority of missense mutations and comprises 10–15% early-onset familial AD between 40–50 years of age [96]. APP is degraded into different product lengths of amino acids APP563, APP695, APP714, APP751, and APP770 isoforms [97]. APP695 is the predominant isoform in neurons is confined to the central nervous system and a larger part of early-onset familial AD mutations are responsible for elevated Aβ42 comparatively to Aβ40 in the brain [98],[99].

Presenilin-1 (PSEN 1) gene with the chromosomal location on 14q24.2 codes for membrane protein γ-secretase complex [100]. 18–50% autosomal dominant early-onset familial AD in 25–65 years age group is most commonly caused by PSEN1 missense mutation suppressing γ-secretase activity with an increased ratio of Aβ42 over Aβ40 accelerates dementia [101],[102]. PSEN1-L166P mutation induces a high rise in Aβ42 production associated with defective intracellular notch signaling domain [103].

Presenilin-2 (PSEN 2) gene is located on chromosome 1q42.13 codes for aspartyl-protease γ-secretase [104]. Rare PSEN2 missense, point, and substitution mutations cause early-onset familial AD affecting high variable age groups between 45–88 years [105]. Studies in human and mice models reported PSEN2 mutations producing fewer rates of Aβ42/Aβ40 in the neurons in contrast to PSEN1 [106].

APOE gene with chromosomal location 19q.13.2 includes gene clusters APOC1, APOC2, APOC4 with three allelic genotypes APOE ∈2, APOE ∈3, and APOE ∈4. APO ∈4 genotype mediates Aβ deposition, hyperphosphorylation of tau linked with high risk for developing early-onset AD, even associated with trauma-stroke reported in humans and transgenic mice [107]–[110]. APOE is involved in cholesterol metabolism, APOE ∈4 enhances amyloid aggregation by receptor-mediated endocytosis, induces cholesterol efflux from neurons and astrocytes. APOE ∈3 isoform has a high affinity for binding to Aβ compared to APOE ∈4 [111],[112]. Patients with AD most commonly display APO ∈4 based mitochondrial deterioration, amyloid plaques, and NFTs pathologies compared to other APOE allelic forms. APOE ∈4 allelic form has a major prevalence for late-onset AD above 65 years [113],[114].

### 3.3. Role of orexin receptors, anxiety, depression, sleep-deprivation, & traumatic brain injury associated with dementia in AD

Orexins—Orexin-A (OR-A), Orexin-B (OR-B) are hypocretin neuropeptide ligands secreted majorly from the lateral hypothalamus, also secreted from locus coeruleus, tuberomammillary nucleus, paraventricular nucleus, and raphe nuclei regions of the brain that binds and activates G-protein-coupled receptors (GPCRs), Orexin receptor-type-1 (OX1R) and Orexin receptor-type-2 (OX2R). Orexin receptors mediate multiple molecular signaling mechanisms connected to feeding behavior, circadian rhythm, energy homeostasis, and drug addiction, sleep disorders, depression, dementia, ischemic stroke, and associated with the pathogenesis of AD [115]–[119]. In AD, the impaired cholinergic pathway alters the sleep-wake cycle with insomnia at night and excess sleep in the day, OR-A increases Aβ42, P-Tau levels in the Cerebro Spinal Fluid (CSF) causes rapid eye movement sleep, orexinergic neurons in the hypothalamus degenerate, and cognitive function deteriorates [120]–[122]. Neuropeptide 26RFa (QRFP), an endogenous ligand of the human orphan receptor GPR103 and GPCRs expressed in the hypothalamic nuclei of the brain executes the same function as orexins. GPR103 correlates with 48% protein sequence homology with OX1R, 47% homology with OX2R. GPR103 forms a functional heterodimer with OXRs signaling cascade function, closely associated with potential neuroprotective effects in AD [123]. The expression and signaling pathways of QRFP/GPR103 are yet to be understood.

Alterations in sleep with deprived sleep quality less than 6 hours and excessive sleep above 9 hours are associated with impairment in cognition [124],[125]. Globally American Academy of Sleep Medicine classifies sleep disorders into six major categories which are linked to having a high risk of causing AD [126]. The first group is insomnia, a depressive condition with difficulty falling asleep associated with the pathogenesis of AD by inducing tau aggregation, amyloid-beta accumulation, neuroinflammation and decreases brain-derived neurotrophic factors (BDNFs) [127]. Secondly, Sleep-related breathing disorders with obstructive sleep apnea (OSA) is a potential risk factor in AD characterized by oxygen-deprived hypoxic repetitive paused breathing, anxiety, fragmentation of sleep, depression, excess day time sleep with naps, the prevalence for OSA is over 70% in AD [128]. Sleep-related hypoventilation and hypoxemia disorders are other sleep-related breathing disorders that altogether promote AD pathogenesis [129]. Thirdly, Central Disorders of Hypersomnolence (CDH) with narcolepsy type 1 & 2, a condition with heavy drowsiness with daytime naps and Idiopathic hypersomnia, a chronic neurological disorder with unsatisfying sleep even after a full night sleep. Kleine-Levin syndrome, a type of rare CDH syndrome with repeating episodes of excessive sleep up to 20hrs a day with behavioral and cognitive anomalies is associated with AD [130]. The fourth group of sleep disorders includes disturbances in circadian rhythm inducing sleep-wake disorders with stress, depression, anxiety, and modification in the melatonin hormonal secretion linked with AD [131]. Parasomnias are the fifth type characterized by abnormal behavior, anxiety, agitation, emotional breakdown, dream with hallucination, falling asleep, sleep disturbances, rapid eye movement (REM) with undesirable body movements, walking or talking during sleep. Lastly, Sleep-Related Movement Disorders with symptoms include drowsiness, clumsiness, confusion, irritability, instability with imbalance; tremor, ataxia are related to cognitive dysfunction, dementia, and PD [132].

Healthy subjective sleep duration is optimal between 6–8 hrs, reported in normal adults [133]. Altered short sleep less than 6hr, prolonged sleep more than 9 hr, and excess daytime sleep with naps are sensitive and early signs of sleep disruption in turn are associated with cognitive decline and dementia [134],[135]. The assessment of subjective sleep in AD patients or caregivers reported anxiety, elevated sleep disturbances, and the abnormal circadian rhythm of sleep-wake patterns, high rated daytime short sleep with naps [136]. Wrist actigraphy analysis of objective sleep on human rest and activity cycles confirmed altered sleep disruption with less total sleep time and a high number of awakenings in AD patients [137]. Studies using Polysomnography (PSG) which reported impaired brain waves signified a high rate of sleep awakening and reduction in total sleep time in AD patients [138].

Electroencephalogram (EEG) detects electrical activity of the brain about communication between neurons via electrical impulses that are active all time, even during sleep. EEG studies in AD patients revealed impaired functional connectivity between frontoparietal and frontotemporal regions of the brain with the reduced slow-wave activity associated with REM sleep anomalies correlated with damaged cholinergic circuit and cognitive impairment [139]. Melatonin hormone released by the pineal gland in the brain at night is associated with the biological rhythm of the sleep and wake cycle, the hormonal release is altered in early AD stages. The CSF-melatonin levels were reported to be declined in preclinical stages of AD associated with sleep deprivation, anxiety, neural degeneration, and cognitive dysfunction [140].

Traumatic brain injury (TBI) causes neurovascular injury with cerebrovascular damage of capillaries, arterial stiffness, perivascular accumulation, damage of blood-brain-barrier, endothelial and mitochondrial dysfunction [141]. TBI is associated with AD-like pathologies inducing Aβ aggregation; hyperphosphorylation of tau with amyloid plaques, NFTs mediated cognitive impairment, and encephalopathy [142],[143]. Clinical biomarkers with tau, p-tau, Aβ were reported in the CSF caused by TBI, indicative of AD. Neuroimaging diagnostic analysis using MRI revealed cerebrovascular damage with micro bleeding, impairment of blood flow in the BBB, and hypo-perfusion produced by TBI. Positron Emission Tomography (PET) scan also exposed accumulation of Aβ plaques, NFTs in the brain accelerated by TBI signifying AD pathologies with cognitive impairment [144].

### 3.4. Challenges in the differential diagnosis of dementia and functional connectivity of the brain associated with AD, DLB & FTD

In some cases of Dementia with Lewy Body (DLB), Aβ-plaques and NFTs co-exist along with α-synuclein oligomers and fibrils with more synaptic loss; the challenge is in discriminating Lewy body dementia from AD [145]. Some of the morphological and clinical studies of the brain help in diagnosing patients with DLB and AD, respectively. AD patients exhibited decreased α-synuclein levels in the CSF and elevated Aβ-plaques, NFTs, and increased neural loss [146]. MRI studies of the brain in patients with DLB exhibited small gray matter atrophy; cortical thickness modification in the posterior parietal lobe of the brain and regional thinning was restricted to lateral frontal, the superior temporal occipital region with less severe hippocampal atrophy [147],[148]. MRI of patients with AD displayed cortical thinning in the subgenual cingulate region, para-hippocampal, and tempo-parietal cortices of the brain with severe hippocampal atrophy affecting the subiculum region, CA1, entorhinal cortex, or hippocampus [149],[150]. Patients with DLB are clinically diagnosed with increased cholinergic dysfunction by gray matter atrophy in the substantia innominate and dorsal mesopontine region of the brain distinguishing from patients with AD [151]. In the early stages of DLB, the damage was diagnosed in the white matter parietal-occipital regions of the brain which is not observed in AD [152]. Studies by functional Magnetic Resonance Imaging (fMRI) in patients with DLB showed enhanced neural connection in the putamen and inferior parietal cortical regions of the brain and declined functionality in the frontal-parietal operculum, the medial prefrontal cortex. Comparatively, the fMRI examination in AD patients exhibited many complex brain network mechanisms with decreased connectivity in the hippocampus, increased prefrontal activity, decreased mesial temporal lobe activation, impairment in memory coding default network between the lateral parietal, temporal, prefrontal, precuneus, posterior cingulate, and medial, and cortical regions of the brain [153],[154].

Imaging a Dopamine transporter with 123I-FP-CIT radiotracer-based single-photon emission tomography (FP-CIT-SPECT) is one of the sensitive and specific diagnostic tools in assessing the dopaminergic function [155]. The dopamine transporter uptake declines in the basal ganglia of the brain cause DLB with dopaminergic dysfunction which is differentiable in contrast to AD [156],[157]. The radiopharmaceutical 123I-meta-iodobenzylguanidine (123I-MIBG) made from Iobenguane, an aralkyl guanidine analog of adrenergic neurotransmitter nor-epinephrine, acts as an antagonist in blocking adrenergic neurons. 123I-MIBG cardia scintigraphy is one more diagnostic tool used in assessing the cardiac postganglionic sympathetic degeneration in DLB cases, but DLB patients additionally diagnosed with cardiovascular heart diseases and diabetes have unveiled fallacious results [158],[159].

Patients with DLB have displayed additional pathologies of amyloid deposits, NFTs, along with α-synuclein protein aggregates. The radioligand biomarker [11C]-Pittsburgh compound B ([11C]PiB) in PET is employed for examining amyloid plaques. Hypo-retention of ([11C]PiB) is observed in patients with DLB in contrast to AD patients, as the amyloid deposition load is excess in AD state [160]. PET imaging with radioligand Fluorine 18-labeled (^18^F) AV-1451 biomarker exhibited excessive uptake in assessing tau and NFTs deposits in the precuneus and temporal gyrus regions of the brain in cognitively impaired AD patients. Meanwhile, (^18^F) AV-1451 uptake in the temporal lobe of the brain was decreased in DLB patients [161]. Montreal Cognitive Assessment (MoCA) was reported to be one of the efficient diagnostic methods to examine cognitive impairment. Observation on visual perception, spatial relationships of objects, task orientation, language fluency, long-term semantic memory, and short-term memory showed a substantial decline in frontotemporal dementia (FTD) patient groups compared to AD [162].

## 4. Biomarkers and therapeutic approaches in AD

Evaluation of Aβ42-biomarker in the CSF using ELISA and Mass Spectrometry has been reported to decrease Aβ42 levels due to sequestration of senile plaques in AD patients [163]. *In vivo* amyloid investigation has also been conducted using PET with diverse amyloid tracers like ^11^C-PiB, ^18^F-AV1451, ^18^F-florbetapir (Amyvid), ^18^F-flutemetamol (Vizamyl), and ^18^F-florbetaben (Neuraceq) [164],[165]. Other CSF biomarkers like—Total tau (T-tau), phosphorylated-tau (P-tau), Chitinase-3-like protein-1 (CHI3L1), Visinin-Like Protein (VLP-1), Neuro Filament Light Protein (NFL), Heart Fatty Acid Binding Protein (HFABP), Neuron-Specific Enolase (NSE), and blood biomarker—Plasma T-tau are shown to be elevated in patients with AD [166]. Tau PET, neurofilament light, and neurogranin are used as new biomarkers in clinical trials to study tau pathology in AD patients [167]. To date, there is no cure for AD and only a few medications can control depression and symptoms that may occur as the disease progresses [168]. US-FDA-approved drugs such as donepezil, galantamine, rivastigmine and tacrine act as cholinesterase inhibitors slowing down the metabolic breakdown of acetylcholine by improving communication between the neural cells and reducing the progression of cognitive impairment proved effective for some patients in the early to middle stages [169]–[171]. Memantine has shown efficacy by acting as a non-competitive NMDA receptor antagonist protecting neurons against excess amounts of glutamate, a messenger chemical released in large amounts to cell-surface NMDA receptors which cause neurodegeneration [172],[173]. Antipsychotic drugs are moderately useful in reducing aggression and psychosis with dementia [174]. These drugs are also associated with adverse metabolic side effects such as cerebrovascular events movement difficulties, cognitive decline that does not permit their routine use [175],[176]. When used in the long-term, they have been shown to associate with increased mortality [177]. γ-secretase inhibitors such as semagacestat, avagacestat, tarenflurbil showed low brain penetration, exhibited side effects associated with cognitive decline in daily activities, increased rates of infections, and skin cancer in AD patients [178]–[180].

Verubecestat, the first small molecule BACE1 inhibitor after long-term treatment in animals reported efficient BBB permeability effectively decreased Aβ40, Aβ42, sAPPβ in CSF, and the brain. Preclinical treatment studies of verubecestat in rats and monkeys did not report adverse effects, such as reduced nerve myelination, neurodegeneration, altered glucose homeostasis, or hepatotoxicity, seen in BACE1-null mice [181]. Unexpectedly administration of verubecestat in patients with early-onset AD caused slight shrinkage in hippocampal and total brain volume which resulted in worsening of cognitive symptoms and other side effects of changes in hair color due to inhibition of BACE2 for its control of hair pigmentation, thus verubecestat was discontinued due to lack of efficacy [182]. Upon oral administration of small-molecule BACE1 inhibitors like lanabecestat, atabecestat in AD patients displayed reduced Aβ, total tau, and phosphorylated tau levels in the CSF, but the usage of these drugs is terminated due to side effects of skin rashes, liver toxicity, and neuropsychiatric symptoms [183],[184].

Due to the inefficacy of drugs in crossing the BBB, different nanotechnology-based drug delivery systems with metal-based silver nanoparticles, gold nanoparticles, polymeric nanoparticles such as poly lactic-co-glycolic acid, polylactic acid, poly butyl cyanoacrylate, polysorbate-80, Cholesterol, DPPC, Methylcellulose, dimethyl-β-CD, sodium taurocholate mediated liposomes are explored in the trial. Using these versatile encapsulated delivery systems; galantamine, curcumin, dexibuprofen, rivastigmine drugs are tested on model systems like Neuro2a cells, HeLa cells, SH-SY5Y cells, GI-1 glioma cells, *in vivo* mice, PC12 cells, bEnd3 cells, glial cells, APPswe/PS1dE Mice, Tg2576 mice, and Balb-C type mice. Conclusively, the advantage of drugs to cross the BBB was easily achieved in vivo with uninterrupted drug delivery to the brain exhibiting inhibition of AchE, cleared amyloid plaques, restricted neurodegeneration, and established neuroprotective effect by improving memory and cognition [185]. A potent pro-apoptotic member; Tumor Necrosis Factor Ligand Superfamily member-10 (TNFSF10) mediates neuroinflammation and Aβ-induced neuronal death. Therapeutics using the anti-TNFSF10 antibody in triple-transgenic-AD (3xTg-AD) mice showed declined neuroinflammation and neurodegeneration in the brain hippocampus [186].

Reports evidenced for higher levels of Angiotensin-Converting Enzyme (ACE) protein expression in the brain with deposition of amyloid-beta and degeneration of hippocampal neurons. Treatment with an ACE inhibitor like captopril has revealed successful regeneration of hippocampal CA1 neurons and reduction of amyloid plaques in the hippocampus of Tg2576 AD mice [187]. Bilateral transplantation of neuronal stem cells in an aged 3xTg-AD restored cognitive and synaptic deficits producing high levels of BDNF without modifying both amyloid plaques and tangle pathologies [188]. Neuronal stem cells that deliver disease-modifying proteins survive for a long period by secreting Aβ-degrading enzyme, neprilysin (NEP) leads to declined Aβ pathology and enhanced the synaptic connectivity in 3xTgAD and Thy1-APP transgenic AD mice models [189]. The Human CNS stem cell line derived from fetal brain tissue has been shown to recover cognitive function by improving the synaptic connectivity in both 3xTg-AD and CaM/Tet-DTA models via growth-associated proteins without affecting Aβ and tau pathology [190]. The therapeutic approach demonstrated in transgenic AD mice models by neuronal stem cells in enhancing neurotrophic factors for improving synaptic activity and delivery of disease-modifying proteins for reducing Aβ pathology could become one of the promising future aspects to combat AD [191]. Early diagnosis of AD using several CSF biomarkers is useful but the pharmacological approach to treat AD is beneficial only in mild to moderate conditions. FDA-approved drugs do help for reducing the symptoms in moderate to severe conditions up to a certain extent; there is a gap in disease-modifying clinical and therapeutic approaches as the disease remains incurable as it advances [192]. The drugs, suvorexant-first dual orexin blocker to treat insomnia and Lemborexant (in phase-3 trials), almorexant, daridorexant, filorexant are some promising dual antagonists of orexin-OX1 and OX2 receptors associated with relieving neuropsychiatric symptoms of sleep-wake disorders and insomnia in AD [193]–[196]. *In silico* evaluation revealed five ligands for site-specific targets on blocking serine protein kinase p-O ester scaffolds of tau with high pharmacokinetics and revealed to be one of the promising therapeutic approaches as p-tau inhibitors for tauopathies in AD [197].

## 5. Healing impact of Physical exercise along with neuromodulative treatments in AD

### 5.1. Molecular role of different forms of Physical exercise in AD

**Figure 5.**
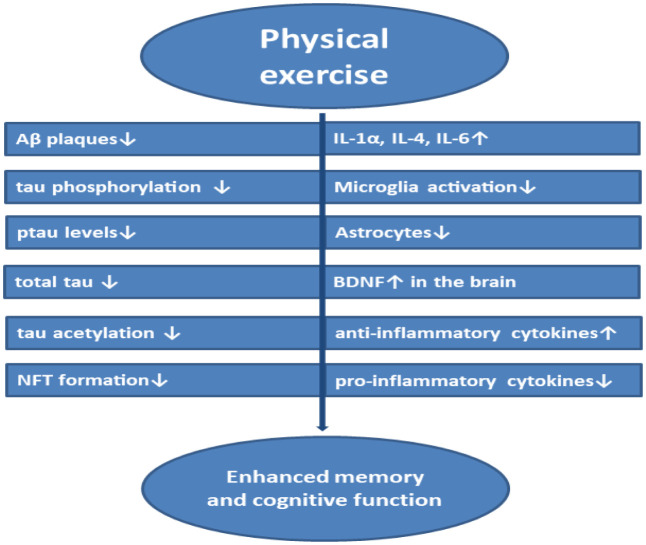
Molecular effects of Physical Exercise in AD for improving memory and cognition.

Physical exercise (PE) has a beneficial effect in decreasing blood pressure, raised endothelial function, improves memory with cognitive psychological function, decreases anxiety and depression enhancing sleep quality by expressing neurotrophic factors inducing neurogenesis [198],[199]. PE has a positive impact on molecules such as PKC, MAPK, Akt, NF-κB, calmodulin kinase, and calcineurin which are involved in antioxidant defense mechanism and cytoprotection through signaling pathways [200]. Animal studies using intra-hippocampal β-Amyloid infusion in Male Wistar rats subjected to one-time treadmill aerobic running and anaerobic strength exercise session exhibited consolidated object recognition learning when examined at different time interval memory tests [201]. Performing physical activity release excess IGF-1 to the brain cells to prevent neurodegeneration. Treadmill running exercise has reported reduced neurodegeneration with enhanced uptake of circulating IGF-1 in different experiments involving excitotoxin domoic acid injected C57BL/6 male mice which produce partial neural loss in hippocampus, adult male Wistar rats injected with neurotoxin 3-acetylpyridine which damages neurons in the brain stem, and pcd mouse inheriting degeneration in the Purkinje cells of the cerebellum [202]. Active PE reduces Aβ induced neuroinflammation in the functional tissue of the brain by clearing Aβ deposits through interstitial fluid drainage by upregulated Aβ transporters [203]. PE in adults with MCI has reported a reduction in tau levels in the CSF [204]. Physical activity controls oxidative stress-induced by vascular risk factors induce cerebrovascular neuroinflammation and neurodegeneration with improved neuron function [205]. PE helps in anti-inflammatory mechanisms by upregulating the expression of IL-10 anti-inflammatory cytokine and downregulating TNF-α, IL-1β pro-inflammatory cytokines [206]. Treadmill exercise in Swiss mice, Balb/cJ, Balb/c-IL4^tm2Nnt^/J knockout mice has revealed reduced microglial activation and an increase in IL-4 cytokines and M2-macrophages which secretes anti-inflammatory cytokines with a decrease in pro-inflammatory cytokines secreting M1-macrophages count reducing the risk of neuroinflammation [207]. The decreasing impact of Aβ, tau phosphorylation, tau levels, NFTs, pro-inflammatory cytokines, microglia, astrocytes, and the increasing impact of anti-inflammatory cytokines (IL-1α, IL-4, IL-6), BDNF, and other factors by PE to enhance cognition is explained in Figure 5.

**Table 1.** Impact of Physical Exercise (PE) on modulating Alzheimer' disease pathologies in different model organisms.

Author (Year)	Model	Test	Molecular alterations by PE
Adlard et al. (2005) [211]	TgCRND8 mice	Morris water maze experiment	Aβ↓ in frontal cortex, hippocampus and improved spatial learning
Um et al. (2011) [212]	Tg-NSE/PS2m mice	Treadmill exercise	Aβ↓ in the brain and improved learning
Leem et al. (2009) [213]	Tg-NSE/htau23 mice	Mouse tread mill	ptau levels↓ in hippocampus, phospho-PKCα↑ phospho-AKT↑ phospho-PI3K↑ phospho-PKA↓ phospho-ERK↓, GSK3β↑
Belarbi et al. (2011) [214]	THY-Tau22 transgenic model	Running wheel test	NFT formation↓ in the hippocampus and enhanced spatial learning
Pajonk et al. (2010) [210]	Human	Aerobic exercise training (cycling)	MRI revealed increased hippocampal volume correlating with neurons and synapses↑, improved neural plasticity
Liu et al. (2020) [208]	3xTg-AD mice (B6; 129-sen1tm1 Mpm Tg (APPSwe, tauP301L) 1L fa/MmJax)	Resistance training, open field test, Novel object recognition test, Y-maze test	expression of pan tau↓ Aβ deposition↓ tau hyperphosphorylation↓ total tau↓ improved cognition, decreased neuroinflammation in the frontal cortex and hippocampus
Alkadhi et al. (2017) [215]	Adult male Wistar rats (infusion of Aβ 1–42)	Rodent treadmill	Increase in APP, BACE-1 and Aβ prevented in the hippocampus, BDNF ↑ in thebrain
Bobinski et al. (2018) [207]	Swiss mice, Balb/cJ, Balb/c-IL4^tm2Nnt^/J knockout mice	Treadmill	Microglia activation ↓ anti-inflammatory cytokine IL-4 ↑ anti-inflammatory cytokine secreting M2-macrophages ↑pro-inflammatory cytokine secreting M1-macrophages ↓
Marquez et al. (2015) [209]	Human	Cycling	BDNF↑ in the serum
Hashiguchi et al.(2019) [216]	APP/PS1 double transgenic mice	Resistance exercise OF test	microglia↑ Aβ plaques↓ IL-1α↑ IL-4↑ IL-6↑ Improved neural function
He et al. (2017) [203]	C57BL/6J mice Thy1-GFP transgenic mice	Voluntary Wheel Training Morris Water Maze	Astrocytes↓ Microglia↓ Glymphatic clearance of Aβ↑ postsynaptic density protein (PSD95) ↑ dendrites↑ unaltered BBB permeability
Mankhong et al. (2020) [217]	Rats	The Single Pellet Reaching (SPR) Test. Rotarod Test The Radial Maze Test Treadmill Aerobic Exercise Training	tau↓ inhibited tau modification and tau acetylation, phosphorylated glycogen synthase kinase 3-beta Tyr 216 (p-GSK3β Y216) ↓ SIRT1↓ Improved memory and cognitive function

Meanwhile, studies using 3xTg-AD mice by resistance training, an examination by novel object recognition, open field-test reported decreased hyperphosphorylation of tau, total tau, and declined Aβ deposits in the hippocampus and frontal cortex of the brain with improved cognition [208]. Trials using humans conducting cycling aerobic exercise showed increased hippocampal volume determined by MRI studies correlated by an increase in BDNFs [209] and elevated density of neurons, with ameliorated synaptic plasticity [210]. A detailed effect of physical exercise in different model organisms and the molecular changes with relevance to AD is explained in Table 1.

PE influences the pharmacokinetics of the drugs suitable for absorption, metabolism, and distribution through oral, subcutaneous, intramuscular, and transdermal ways by altering physicochemical characteristics like pH of enzymes in the gastrointestinal tract, pH of the blood and muscle. PE also induces total body clearance of drugs and increases renal and biliary excretion, avoiding drug toxicity [218].

WHO recommends health guidelines on performing the physical activity for aged people above 65 years at least 150 mins-moderate/75 mins-heavy or equivalent physical activity daily with short-10mins intermediate breaks. Considering added health benefits, daily 300 mins-moderate/150 mins-heavy or an equivalent physical activity is recommended. Aged individuals with poor mobility are suggested to do mild balance exercises thrice a week and any kind of muscle-strengthening physical activity, twice a week. The elderly adults with any health complications and incapable of performing the recommended activities are advised at least to remain active physically, depending on the individual capabilities [219]. 2020 WHO physical activity guidelines describe that adult age groups between 18–64 years are recommended with 150–300 mins-moderate/75–150 mins-heavy physical activity and categorizes different forms of physical activities as i) aerobic physical activity including yoga, meditation, walking, running, swimming, and bicycling, ii) balance training exercises with different postures, iii) muscle and bone-strengthening exercises with treadmill running, gymnasium, weight lifting, iv) Household-domain physical activity, v) Leisure-domain physical activity with sports, dancing and gardening, vi) Light-intensity physical activity (LPA) with slow walking, bathing, or other incidental light activities at a normal breathing rate. [220].

Physical activities directly or indirectly have a major impact on relieving obesity, stress, sleep deprivation, cardiovascular diseases, and improve hippocampal neurogenesis, induce synaptogenesis, enhances BDNF with cognition reported both in human and rat models [221]–[223]. Aerobic exercise promotes beneficiary structural and functional changes in hippocampal size, reduced loss of grey, white matter in the frontal and temporal regions of the brain observed by voxel-based morphometry (VBM), MRI significantly improved neuroplasticity with spatial and learning memory in humans [224],[225]. A scheduled 3–12 months physical aerobic exercise training in aged individuals showed enhanced neural connectivity with improved cognition, memory, reduced anxiety, stress, and depression [226],[227]. A study using analyzing physical exercise in 198 subjects with MCI and 1,126 with normal cognition reported conservative activity in the mid-age and 39% lower risk of developing MCI by aging [228]. Physical activity with aerobic exercise enhanced cognition, independent functioning, learning, and memory in older adults with MCI and dementia [229]. PE with aerobic multicomponent training in AD patients displayed reduced neuroinflammation, ROS, improved cognition analyzed by neuropsychological battery test [230],[231]. Physical activity with walking, stretching balance exercise for 3-sessions a week till 3-months decreased depression, better mood, increased behavioral response, and cognitive function in early to moderate-AD patients [232],[233]. Stationary bike aerobic arms cycling for 20 mins thrice a week for 3-months at 70% maximal heart rate showed enhanced attention, response to verbal communication with improved cognitive thinking [234]. Synergistically, multiple antioxidant-rich healthy diets with regular physical activity in aged peoples could decrease the risk for dementia by reducing ROS, mitochondrial dysfunction, and other disease pathologies associated with MCI, AD and improve neurocognitive function [235].

### 5.2. Combined role of Neuromodulatory treatment-with physical exercise in AD

Repetitive Transcranial Magnetic Stimulation (rTMS) is a neuromodulation instrument treatment technique that involves electro-magnetic impulses delivered through coils applied on the forehead as helmet helps stimulate synaptic transmission in the focal cortical regions of the brain. rTMS values with a repetitive minimum frequency below 1 Hz with the continuous form of theta-burst stimulation (cTBS) indicate debased low synaptic transmission, high-frequency values between 5–50 Hz intermittent forms of theta-burst stimulation (iTBS) correlates with enhanced cortical excitability in synaptic transmission. rTMS is an FDA-approved treatment procedure for major depression, treatment-resistant depression, anxiety, AD, and psychotic disorders [236]–[238]. rTMS with 20 Hz stimulation on the dorsolateral prefrontal cortex (DFLPC) and precuneus of the brain reported improved episodic memory, language function, identifying objects, comprehensive auditory learning in patients with mild to moderate AD [239],[240]. Performing various cognitive tasks in mild AD patients with mild depression during rTMS procedure with 20 Hz stimulation, 40 pulses per burst in 5-second intervals on the right and left DFLPC region of the brain showed improvement in retained cognition for certain weeks post-treatment [241]. Patients with early AD diagnosed by brain CSF protein levels, shown beneficiary results on delayed recall and enhancement in memory after two weeks of rTMS 20 Hz stimulation on the precuneus region of the brain [242].

rTMS with cognitive training in 30 mild-to-moderate AD patients involving grammar task, comprehensive meaning, attentive spatial memory tasks recognition-categorizing objects, shapes, places, colors, naming letters focusing the Broca area, Wernicke area, left to right DLPFCs and PSAC areas of the brain, with the schedule of 1 h daily, weekly 5-days, for 6 weeks reported safe, effective, improved cognition up to a year in 80% of AD patients [243]. Combined application on performing rTMS with PE in stroke patients has shown a positive impact on improving voluntary functioning with reduced long-term depression, modulates neurotransmitters, induces BDNF generation, and improves neural plasticity [244]. rTMS (18 Hz, 2-Sec on, 18-Sec off) combined with stationary aerobic cycling in patients with depression on continuous assessment of alertness, response, cycling capability reported that all patients were comfortable during rTMS-physical activity proved effective, improved mood with reduced depressive symptoms [245].

Transcranial electrical stimulation (tES) is a safer and reliable technique that noninvasively stimulates the brain by passing electrical current via electrodes through the soft tissue and skull into the brain cortex and alters brain function, also includes multiple types of tES-transcranial direct current stimulation (tDCS), transcranial alternating current stimulation (tACS), transcranial random noise stimulation (tRNS), transcranial pulsed current stimulation (tPCS), have proven beneficiary in inducing cortical excitability and neuromodulator effects [246]. Comparative evaluation on the effects tES types- tDCS, tACS, tRNS, and tPCS in a beta-amyloid-induced AD rat model (Sprague-Dawley male rats bilaterally microinjected with Aβ25–35 dose of 5 µg/2.5 ml/day into brain hippocampus), physical activity(swimming) training with behavior assessment using Morris water maze task and tES induction (20 min-session, current intensity-200 µA, ramp-10 secs in multiple sessions) proved that both tDCS and tACS was most significant in improving learning and memory behavior induced by Aβ [247]. Aerobic exercise with tDCS synergistically enhances BDNFs and other growth factors; releases neurochemicals in the brain improves synaptic activity and motor function with cognition [248].

## 6. Conclusions

Aβ plaques and NFTs are the pathologic hallmarks of AD linked with multi-factorial integrated molecular events. Since the disease progression in the early stages is asymptomatic; fluid biomarkers are boon for early diagnosis of AD. Unfortunately, FDA-approved drugs used in AD treatment could only reduce the symptoms but not the disease progression. Successful use of neuronal stem cells on transgenic AD mice models has shown promising results in both reducing Aβ by secreting Aβ-degrading enzyme, NEP and enhancing BDNF. Treatment with γ-secretase inhibitors, BACE1 inhibitors can strongly cut down Aβ40, Aβ42, total tau, and phosphorylated tau pathologies. Experiments on cycling, treadmill PE in human trials, transgenic AD mice, and rat models appear as the best informative sources in being a preventive measure to avoid developing AD and prove efficient in modulating Aβ, tau, and neuroinflammation in developed AD. The pharmacokinetics in assimilation and absorption of the drugs could be more effective by PE, conjointly the ability of drugs to cross the BBB could be achieved by nanotechnology-based drug delivery systems for degrading amyloid plaques, inhibiting acetylcholine esterase, and preventing neurodegeneration by enhancing neuron function. Conclusively, the joint approaches on using neuronal stem cells with nanotechnology-based multi approached drug delivery systems along with a healthy diet and physically active stress-free lifestyle with neuromodulatory treatments like rTMS, tES may prevent AD pathologies and improve the synaptic transmission of the brain that would become one of the future therapeutic research advancements in tackling AD.
